# Influence and Compensation of Temperature Effects for Damage Detection and Localization in Aerospace Composites

**DOI:** 10.3390/s20154153

**Published:** 2020-07-26

**Authors:** Guillermo Azuara, Eduardo Barrera

**Affiliations:** Instrumentation and Applied Acoustics Research Group, Universidad Politécnica de Madrid, 28031 Madrid, Spain; g.azuara@upm.es

**Keywords:** guided waves, Lamb waves, composite, temperature compensation, damage detection

## Abstract

Structural Health Monitoring (SHM) of Carbon Fiber Reinforced Polymers (CFRP) has become, recently, in a promising methodology for the field of Non-Destructive Inspection (NDI), specially based on Ultrasonic Guided Waves (UGW), particularly Lamb waves using Piezoelectric Transducers (PZT). However, the Environmental and Operational Conditions (EOC) perform an important role on the physical characteristics of the waves, mainly the temperature. Some of these effects are phase shifting, amplitude changes and time of flight (ToF) variations. In this paper, a compensation method for evaluating and compensating the effects of the temperature is carried out, performing a data-driven methodology to calculate the features from a dataset of typical temperature values obtained from a thermoset matrix pristine plate, with a transducer network attached. In addition, the methodology is tested on the same sample after an impact damage is carried out on it, using RAPID (Reconstruction Algorithm for Probabilistic Inspection of Damage) and its geometrical variant (RAPID-G) to calculate the location of the damage.

## 1. Introduction

Non-Destructive Inspection (NDI) is one of the most important disciplines within structural engineering [1], enabling the detection of damage to structures prior to a mechanical failure [2,3]. However, inspection time, operator subjectivity and cost are factors that make NDI only effective to scheduled maintenance. Structural Health Monitoring (SHM) emerges as the structural inspection alternative to online and in-service monitoring of the functional state of the structure [4,5]. Recent advances in signal processing techniques [6] and technology [7] give this field a promising future. One of the most important applications of SHM is, in addition to monitoring civil structures [8,9], rotating machinery [10,11] and the terrestrial transportation industry [12,13], the aeronautical industry [14,15].

In recent years, the manufacture of aircraft parts with Carbon Fiber Reinforced Polymer (CFRP) composite materials has increased to over 50% of their weight [16,17], reducing the total weight of the aircraft, also improving mechanical properties.

The application of SHM techniques on these type of materials, even though it is very advanced, presents several drawbacks, mainly due to the inherent material anisotropic nature, especially when the geometry of the structure is complex, making the SHM real application in an aircraft a difficult challenge [18], in addition to other factors such as the weight of the instrumentation, the difficulties of wiring sensors and the cost-utility analysis of the technology [19].

Since the fundamental axiom of SHM for damage detection is the comparison between, at least, two different signal datasets [20], it is worth mentioning that the most extended solution for damage detection and location is the comparison of pre-damage information (i.e., in the pristine state) with the information of the structure when damaged [21]. However, the unavailability of a baseline dataset recorded at the same temperature at which damage is recorded is unlikely, firstly due to the impracticality of recording a baseline dataset for every operating temperature, and secondly, because a small variation in temperature, under operating conditions, causes large variations in the response of the structure to Lamb waves, producing changes in both the phase and the amplitude of the different wave packets recorded by the sensors [22].

An alternative to the traditional procedure of damage detection (i.e., baseline vs. damaged) is the evaluation comparing two tests in the damaged state conditions (baseline-free), but varying some conditions of the excitation (amplitude of the excitation signal [23], multi-frequency tests [24], etc.). However, since this methodology is based on the appearance of nonlinearities in the structure response (e.g., non-uniform signal’s envelope distribution variation, appearance of higher-order harmonics or changes in phase velocity), the possible inherent nonlinearities of the composite material make the application of this method not suitable to all geometries (e.g., the geometry generates itself nonlinearities in the guided waves). In addition, to make the method more effective, it is necessary to have a physical knowledge of the problem, since the produced nonlinearity depends on the type of damage [25].

In this paper, the SHM traditional point of view was applied, using baseline datasets information, and a data-driven method was used for the evaluation and correction of temperature effects [26], assuming that the temperatures distribution field on the sample was homogeneous [27], as well as based on minimizing the difference between several types of baseline tests, adjusting each of them to a reference temperature and, with the use of polynomial regression, being able to estimate the parameters used for compensating a signal recorded at the current damaged state, at a certain temperature at which the baseline data is not available. Finally, to check the effectiveness of the method applied to damage detection and location, the data from the current state were adapted to several available baselines at several different temperatures, and an artificial damage was detected and located, using an imaging algorithm (RAPID, Reconstruction Algorithm for Probabilistic Inspection of Damage) [28] and, based on previous experience [29], a modification of this algorithm (RAPID-G). The effectiveness of the combination of these two methodologies (both signal reconstruction and imaging) is analyzed in this paper, providing a solution to achieve better damage detection and localization in plate-like structures.

This paper is structured into the following sections: In Section 2, the experimental set-up is described; Section 3 provides the mathematical procedures to evaluate and compensate temperature effects, as well as a brief explanation about the imaging algorithm; in Section 4, the obtained results from ToF calculation, temperature-dependent parameters and correction and the imaging algorithm are described; finally, Section 5 deals with the discussion and conclusions of the work.

## 2. Materials

**Figure 1:**
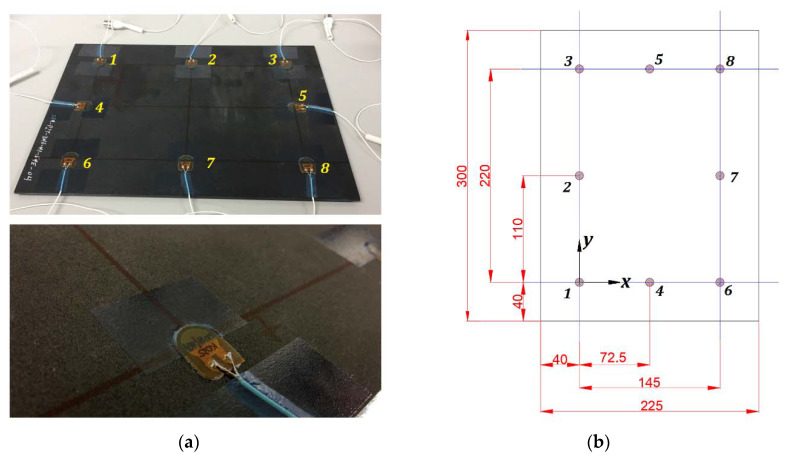
(**a**) At top, sample with transducers attached and numbered. At bottom, detail of the attached transducer. (**b**) Specimen’s dimensions, units in (mm). Transducer number 1 is the origin of coordinates.

In this work, a thermoset matrix CFRP plate was used, whose characteristics are shown in Table 1. The sample and its dimensions are shown in Figure 1. It was instrumented with eight PZT DuraAct transducers, with active disc D10 mm × TH 0.2 mm, from PiCeramic [30], attached to the surface using an epoxy adhesive from DELO (AD821), working both as actuators and sensors.

**Figure 2:**
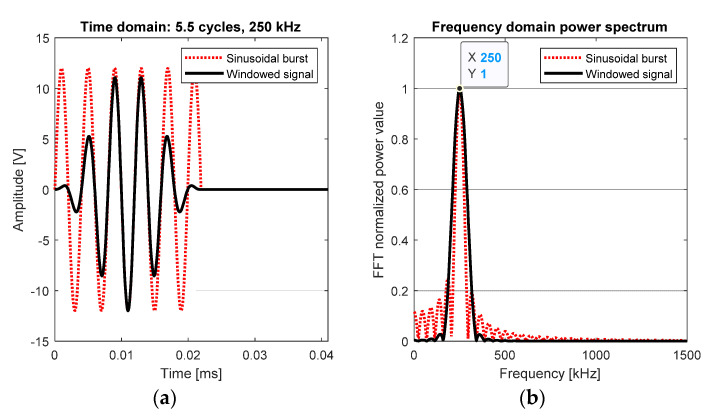
Excitation signal comparison, before (red) and after windowing (black). (**a**) Time domain waveforms. (**b**) Fast Fourier Transform (FFT) analysis.

The type of each test performed (Round-Robin), in pitch-catch configuration, resulted in a full-matrix analysis (8 transmitters × 8 receivers = 64 signals/test). Excitation signals were sinusoidal tone bursts of a different number of cycles and central frequencies, 12 V amplitude. Hann window was used to get a centered-frequency signal, resulting in both narrowband excitation and response in the transducers. The excitation signal’s waveform and its frequency domain are depicted in Figure 2. The data processing was carried out using MATLAB^®^ scripts. The used excitation conditions are in Table 2.

**Figure 3:**
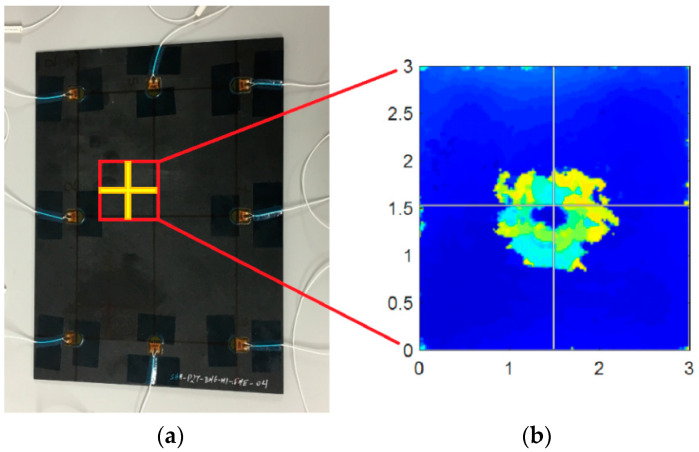
(**a**) Impacted zone, point (*x* = 5.5, *y* = 14.5) (cm). (**b**) C-Scan tomography, units in (cm).

To perform baseline tests at different temperatures, a TAS Series 3 climatic chamber was used, performing tests from −40 °C to 50 °C, in 5 °C steps, resulting in 19 different temperatures datasets (see Supplementary Materials), typical of operation temperatures for in-service aeronautics [31]. For every temperature, two tests at the same excitation were carried out. After recording the baseline data, the sample was impacted using an Instron CEAST 9350 Drop Tower, performing a 20 J-energy impact, using a spherical impactor with a mass of 2.41 kg and 20 mm radius, resulting in a barely visible impact damage (BVID). The subsequent C-Scan analysis allowed to check the damaged area (Figure 3), showing an internal delamination. Following these verifications, a new group of tests was performed, but in this case only at room temperature (in this case, 20.5 °C), with the same characteristics as in Table 2.

## 3. Methods

In the study carried out in this paper, a distinction was made mainly between the following analysis: calculation of the time of flight (ToF) for each transmitter-receiver path, the extraction of temperature-dependent parameters for each path and temperature, a polynomial regression for these parameters as a function of temperature, the correction of the current temperature signals, and imaging for damage detection and location.

### 3.1. Time of Flight Extraction

**Figure 4:**
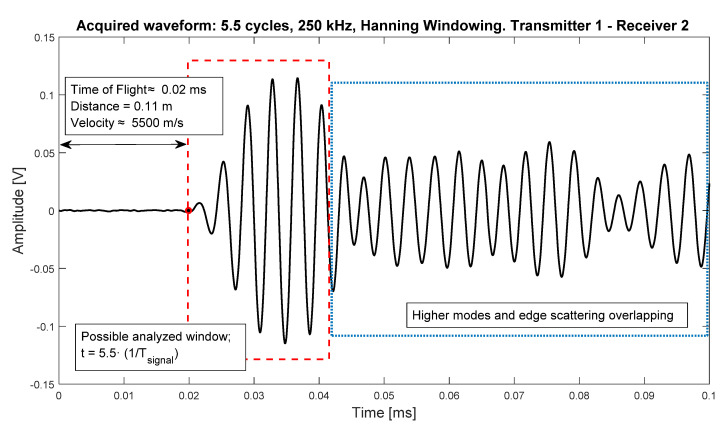
Acquired signal propagated from transducer 1 to transducer 2. The ToF was calculated using cross-correlation method, and the arrival point is on *t* = 0.02 ms. Excitation signal was 250 kHz, 5.5 cycles toneburst.

ToF, which is the time that it takes for the guided wave for travelling from the actuator to the sensor in a pitch-catch configuration, is one of the most important parameters of guided waves propagation, since its knowledge allows to analyze a section of interest of the signal, starting from the arrival point of the wave. Figure 4 shows the point of arrival of the guided wave, an analysis region of interest containing first arrivals information (in this case, 5.5 cycles of the wave, same as the excitation signal) and the region containing useless information due to overlapping modes and wave packets. Moreover, in CFRP, the knowledge of the propagation velocity is very important, since it changes with the direction of propagation due to the anisotropy of this type of materials. In this study, the calculation was made obtaining the time which the cross-correlation (1) between the excitation signal and the acquisition signal is maximum [32]. Using this procedure, the group velocity of the S0 mode was obtained, which is the fastest in these materials, as well as the predominant mode at the frequencies used (250–350 kHz [33]) and, furthermore, the one that best interacts with the internal defects as an impact damage [34]. In addition, and due to the overlap between different modes wave packets, the A0 mode was not analyzed, besides being useless for this study (that mode interacts very well with surface damages [34]). Finally, the average ellipse of the velocity distribution [35] was estimated.

(1) $$\left(x⊗y\right)\left(\tau \right)=R_{xy}=\underset{T\to \infty }{\lim}\frac{1}{T}\int_{0}^{T}x\left(t\right)\cdot y\left(t+\tau \right)dt$$

### 3.2. Temperature and Dependent Parameters Analysis and Correction

**Figure 5:**
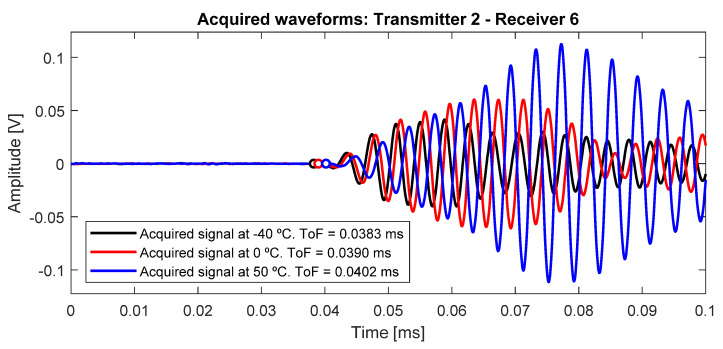
Different signals at different temperatures, in the interrogation path 2–6. The ToF, the amplitude and the instantaneous phase, in this time window, increase with the temperature.

The effects of temperature on the propagation of Lamb waves is determined by its influence on the transducers and the mechanical properties of the structure material [26]. These effects produce the variation, mainly, in the amplitude of the wave packets and the instantaneous phase, affecting their envelope and, therefore, their energy distribution (Figure 5). The envelope is set by the interrogation path between transducers and the geometry and edge boundaries.

To compensate these effects, the analytic complex signal (2) was used, so that both the amplitude and the instantaneous phase of the signal (3) were extracted:

(2) h(t)=x(t)+jH{x}(t),

where h(t) is the analytic complex signal, x(t) is the real (acquired) signal, j complex imaginary unit, and H{x}(t) is the Hilbert transform. The amplitude and phase difference parameters are presented below:

(3) A(T1→T0)=AT1AT0; ϑ(T1→T0)=φT1−φT0.

**Figure 6:**
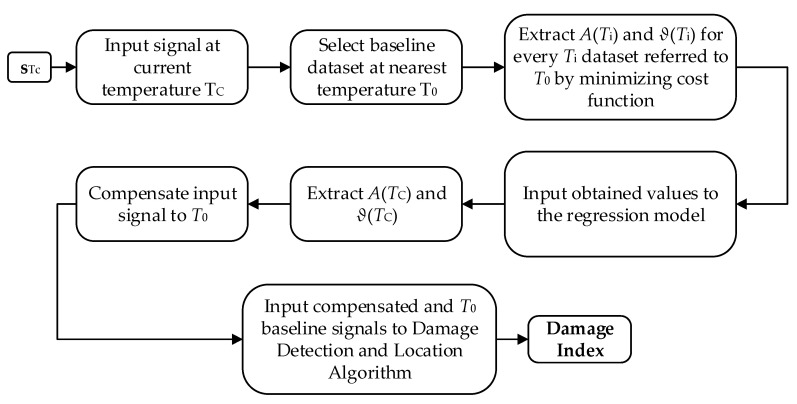
Flowchart of the algorithm.

Finally, to correct the signal at $T_{1}$ temperature to $T_{0}$ temperature, the following operation (4) was carried out [26]:

(4) sT1→T0(t)=Re(A(T1→T0)·h(sT1(t))·e−jϑ(T1→T0)),

where A(T) is the amplitude compensation factor, $\vartheta \left(T\right)=\vartheta_{1}-\vartheta_{0}$ is the phase compensation factor, h(·) is the Hilbert analytic complex signal from $s_{1}$, and Re(·) is the real-part operation, which allows to extract the real signal from the complex Hilbert envelope. Figure 6 shows the steps of the procedure.

### 3.3. Polynomial Regression

**Figure 7:**
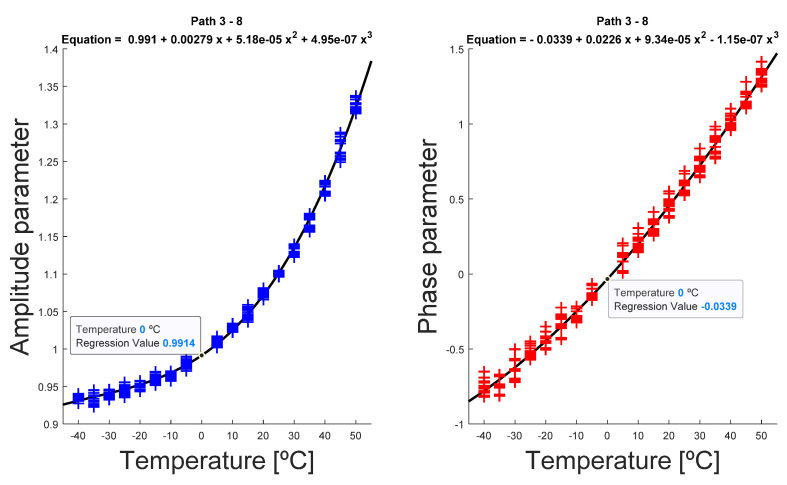
Evolution of temperature-dependent parameters for interrogation path 3–8. In this example, excitation signal was 250 kHz frequency, 5.5 cycles. The reference temperature is *T*_0_ = 0 °C, and the estimated parameters at that temperature are, approximately, $A\left(T_{0}\right)=1$ and $\vartheta \left(T_{0}\right)=0$.

**Figure 8:**
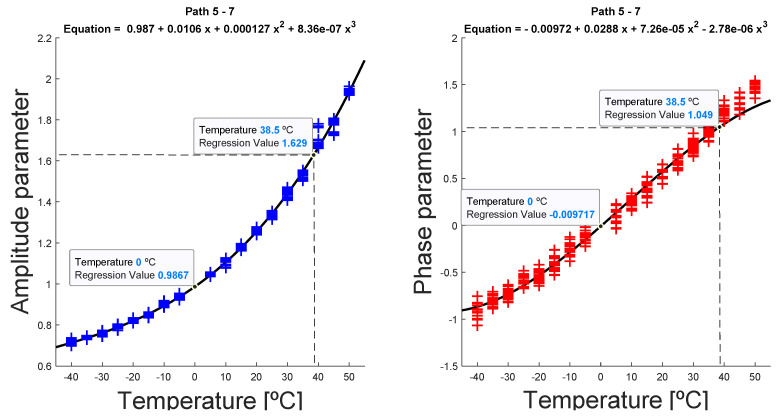
Interpolation and estimation of temperature-dependent parameters, for a reference temperature of *T*_0_ = 0 °C, assuming a current temperature of *T*_c_ = 38.5 °C, for the interrogation path 5–7. In this example, the excitation signal was 350 kHz frequency, 3.5 cycles.

**Figure 9:**
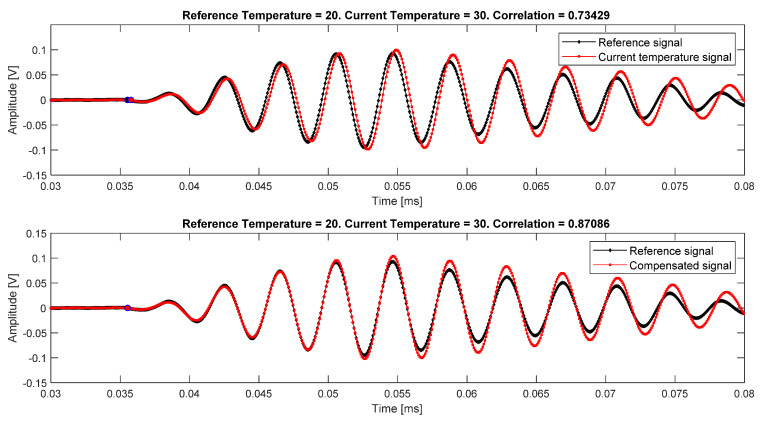
Signals from interrogation path 4–5. At the top, signals before compensation, the correlation coefficient is *ρ* = 0.73. At the bottom, signals after compensation, the correlation coefficient increases up to *ρ* = 0.87. In this example, the excitation signal was 250 kHz frequency, 5.5 cycles.

Since tests were carried out for the pristine state every 5 °C, no pristine data are available for intermediate temperatures. Therefore, the distribution of each of the parameters A(T) and ϑ(T) over the temperature range must be analyzed. Assuming that the temperature of the available damaged test is $T_{c}$, the following procedure was carried out:

1. Select a reference temperature $T_{0}$ at which the $T_{c}$ signal at damaged state must be compensated, preferably corresponding to a baseline dataset recorded at a temperature as close as possible to $T_{c}$, in order to avoid large deviations in the following steps.
2. Perform an iterative process of comparison within the rest of the available baseline datasets at temperature $T_{i},\;i=1,\;2,\ldots 19$, with the reference dataset at $T_{0}$, for all the propagation paths.
3. Extract the correction parameters $A\left(T_{i}\right)$ for every available temperature $T_{i},$ by minimizing the cost function J (5):
  (5) J=∥sT0−sTi→T0∥=∥sT0−Re(A(Ti→T0)·H(sTi(t))·e−jϑ(Ti→T0))∥
  where ∥·∥ is the 2-norm of the difference between reference signal and comparison-baseline signal. The minimization of the potential was made through a least-squares procedure, with $A\left(T_{i}\right)$ and $\vartheta \left(T_{i}\right)$ as variables.
4. Once the amplitude and phase parameters for each temperature are obtained, the polynomial regression for both factors as a function of temperature was carried out, again using a least-squares regression model. In this case, the polynomial grade was adjusted considering the lowest residual value, resulting in most cases in a third-degree polynomial (Figure 7), although on certain occasions a linear polynomial and quadratic polynomial were obtained.
5. For the signals at current temperature $T_{c}$, the parameters were interpolated (Figure 8), obtaining the parameters $A\left(T_{c}\right)$ and $\vartheta \left(T_{c}\right)$, and finally correcting the signal from $T_{c}$ to $T_{0}$ using (4) (Figure 9).

### 3.4. Imaging Algorithm

Due to the chosen distribution of the sensors over the sample, as well as its anisotropy, the RAPID [28] algorithm was chosen for the detection and location of the damage. However, the preliminary results were not as expected, hence, to avoid the influence of the intersection points in the image results, the modification of the algorithm called RAPID-G [29] was used, and finally the effectiveness of each method was assessed.

The standard RAPID is composed of two terms: the elliptical geometrical distribution for each path ($E_{ij}$) and the damage index for each path ($DI_{ij}$) (6). Besides, an additional weighting term ($G_{k}$) is added in RAPID-G modification, which takes into consideration the distance of the analysis point $\left(x_{p},y_{p}\right)$ to every considered *k* intersection point between paths. Finally, the equation states as follows (6):

(6) $$P\left(x_{p},y_{p}\right)=\overset{N}{\underset{i=1}{\sum }}\overset{N}{\underset{j=1,j\neq i}{\sum }}DI_{ij}E_{ij}\left(x_{p},y_{p}\right)\cdot \overset{M}{\underset{k=1}{\sum }}G_{k}\left(x_{p},y_{p}\right)$$

**Figure 10:**
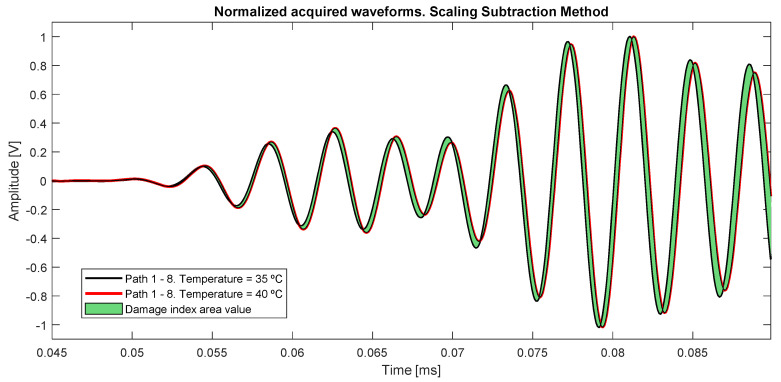
Baseline acquired signals from path 1–8 at different temperatures. The colored area is the value calculated with the SSM damage index, in the case of a comparison between very close temperatures.

The damaged index selected is the Subtraction Scaling Method (SSM) [23], which normalizes the compared signals and calculates the area between them (Figure 10) through integration, in the analyzed time section nT of the signal (7).

(7) $$DI_{ij}=\frac{1}{nT}\int_{0}^{nT}{\left(s_{damaged}\left(t\right)-s_{baseline}\left(t\right)\right)}_{ij}^{2}dt$$

Figure 10 shows the possible incurred error in the case of using signals at close temperatures (ΔT = 5 °C) as the same baseline, obtaining deviation from both amplitude and instantaneous phase.

## 4. Results

**Figure 11:**
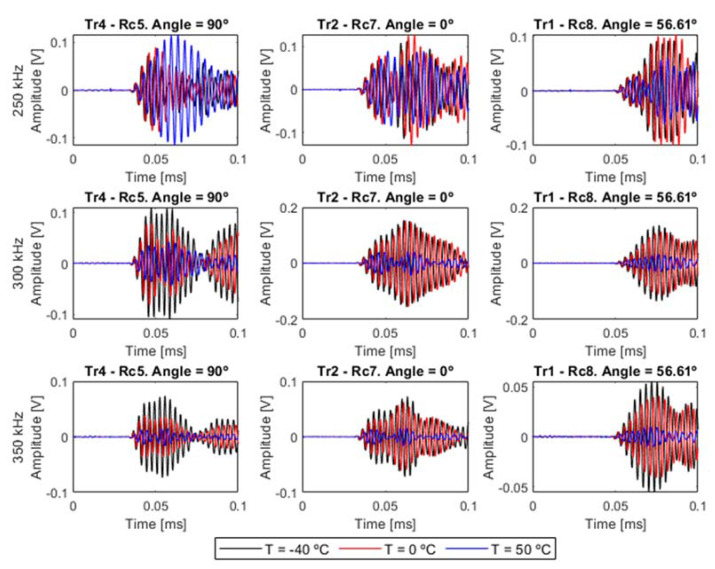
Time domain responses on main directions, at three different frequencies and at three different temperatures. The excitation signal was 5.5 cycles and Hanning windowed.

In this section, the corresponding results to each analysis are presented. As an example, in Figure 11, some system’s responses at some different frequencies and temperatures are plotted. The selected paths were selected from the main directions of the laminate (angles 0° and 90°), and the available diagonal path direction at 56.61° (path 1–8). Figure 11 shows that the evolution of Lamb waves strongly depends on the temperature, which modifies both amplitude and phase, and the selected path angle, whose orientation directly affects the ToF of the different modes and the scattered waves from the edges.

### 4.1. Group Velocity Dependance

**Figure 12:**
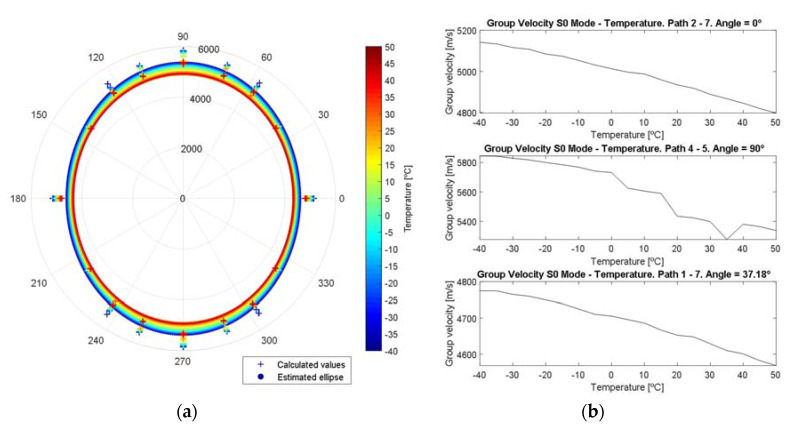
Group velocity dependence with the temperature. (**a**) Polar graph group velocity—angle between transducers. The ellipse’s area decreases as the temperature increases. (**b**) Evolution of group velocity with the temperature, paths 2→7, 4→5 and 1→7.

In Figure 12, the dependence of group velocity is plotted. The analyzed material presents anisotropy in the group velocity, and its evolution with the temperature is almost linear, decreasing when the temperature increases. The ellipse was calculated following the direct least square fitting method described in [35].

### 4.2. Obtained Parameters from Temperature Model, Regression and Compensation

This section is divided into three subsections: the calculation of temperature parameters for certain temperatures, the polynomial regression for estimating the evolution of these parameters with the temperature for each path, and the final assessment of the effectiveness of the correction in the signals.

#### 4.2.1. Calculation of the Temperature-Dependent Parameters

**Figure 13:**
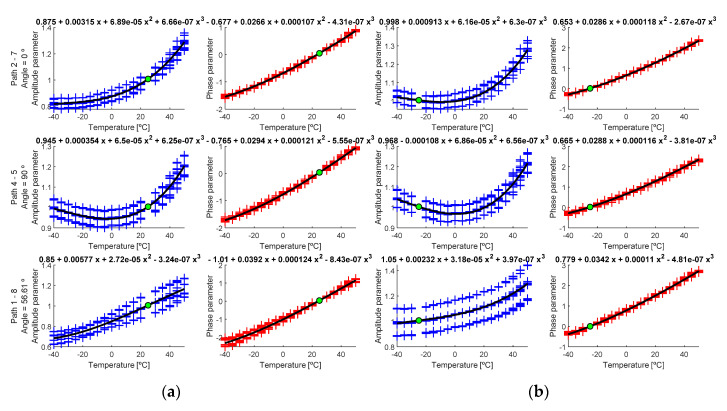
Calculated parameters (cross marks) and regression curve (black line) for (**a**) $T_{0}$ = −25 °C and (**b**) $T_{0}$ = +25 °C. Green point is the estimated point for $T_{0}$. Left *y*-axis shows calculated paths and the angle between transducers.

As mentioned above, the temperature-dependent parameters are essentially amplitude and instantaneous phase. As an example, in Figure 13, the evolution of both parameters for paths in the main directions at 0°, 90° and oblique direction (56.61°) are depicted, and at two different reference temperatures, $T_{0}=25\;{}^{\circ}C$ and $T_{0}=-25\;{}^{\circ}C$. The available dataset made it possible to carry out eight different analysis for each corrected temperature.

As seen in Figure 13, the parameters follow different polynomial distributions. In general, phase parameter increases with the temperature, which means the phase has higher value when the temperature is higher. On the other hand, the temperature value depends on the selected path, since additional scattering effects affect the amplitude (mainly edge reflections). In the following section, the coefficients of regression polynomials are calculated using an average of the signals for each condition instead of every single signal comparison.

#### 4.2.2. Polynomial Regression for the Temperature-Dependent Parameters

**Figure 14:**
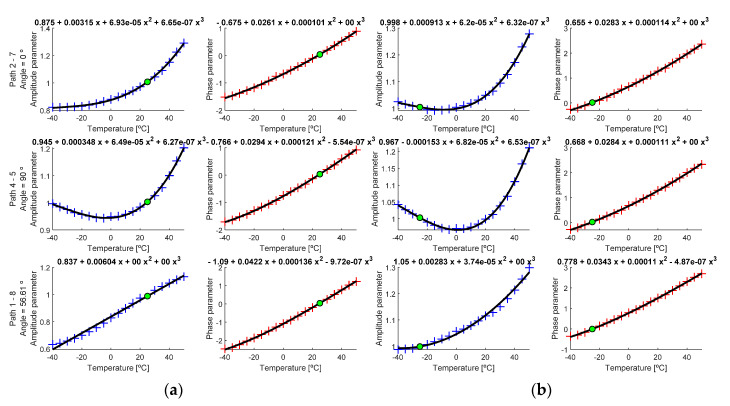
Calculated parameters (cross marks) and regression curve (black line) for (**a**) $T_{0}$ = −25 °C and (**b**) $T_{0}$ = +25 °C. Green point is the estimated point for $T_{0}$. Left *y*-axis shows calculated paths and the angle between transducers.

As mentioned, to perform this analysis, the average of all signals at the same temperature and in the same interrogation path between transducers was calculated, resulting in a more robust data and reducing possible signal noise [20]. The obtained parameters were adjusted to the lowest possible polynomial grade against a threshold value for the sum of the residual values of 10^−2^. Figure 14 shows the evolution of the parameter value obtained from averaged signals, for the same temperatures as used in the previous paragraph.

Once the regression curve was calculated, the parameters were calculated substituting the current temperature $T_{c}$ in the obtained polynomial, calculating this way $A\left(T_{c}\to T_{0}\right)$ and $\vartheta \left(T_{c}\to T_{0}\right)$, and then using (4) to translate the current signal from $T_{c}$ to $T_{0}$, at which temperature a baseline was available.

### 4.3. Damage Detection and Location

#### 4.3.1. Temperatures Selection and Tests Compensation

**Figure 15:**
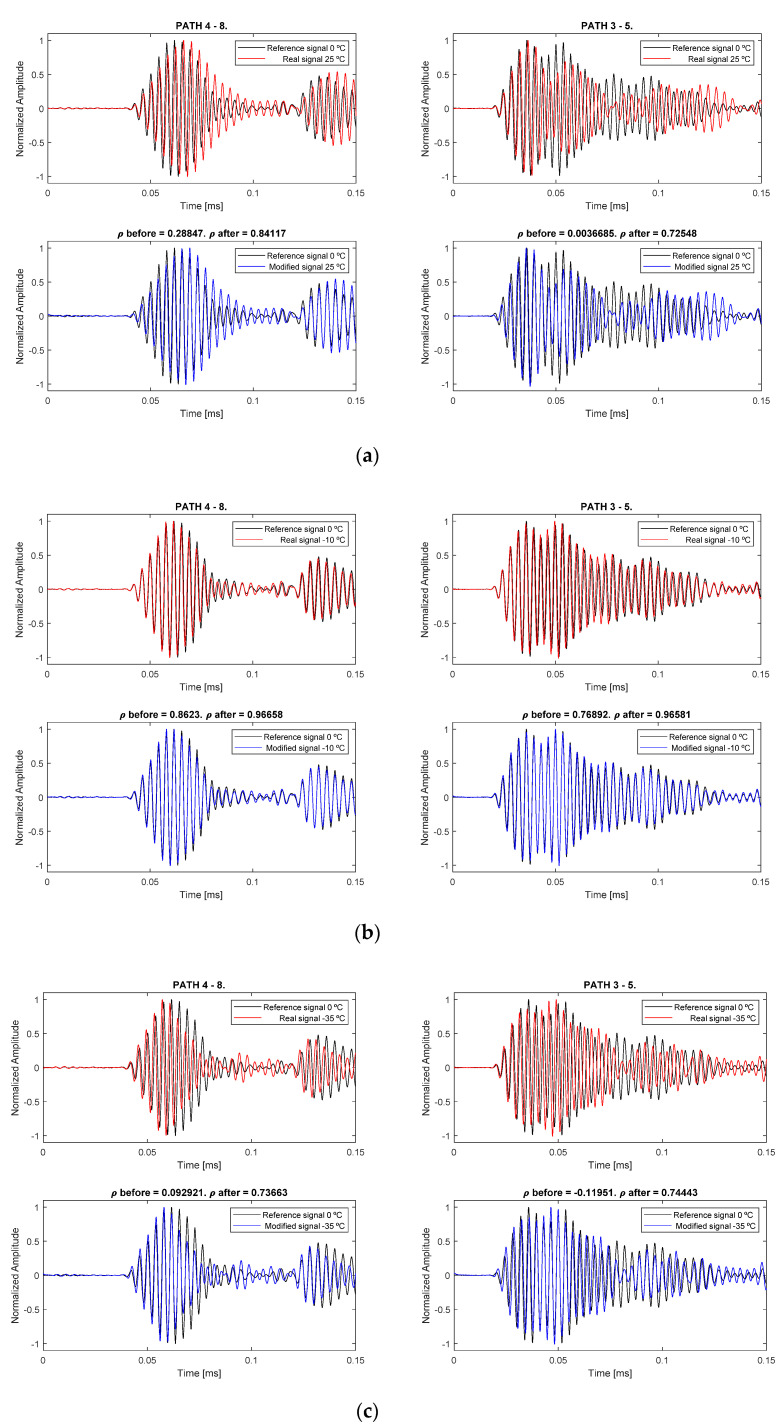
Different examples of signal compensation to 0 °C reference, for two different paths (4–8 and 3–5), from three different real signals to compensate. The first group (**a**) is $T_{c}$ = 25 °C, the second group (**b**) is $T_{c}$ = −10 °C, and the third (**c**) is $T_{c}$ = −35 °C.

Figure 15 shows the correction of some baseline state signals for some representative paths and, as a measurement of reliability, the correlation coefficient *ρ* before compensation and after compensation was calculated. As expected, the closer the current temperature to the reference, the higher the correlation coefficient. However, in every case the coefficient value increases significantly (Δ*ρ* = 0.56 and 0.72 in the first graph, Δ*ρ* = 0.11 and 0.20 in the second graph and Δ*ρ* = 0.64 and 0.86 in the third graph, as described in Table 3).

#### 4.3.2. Damage Detection and Localization Using an Imaging Algorithm

**Figure 16:**
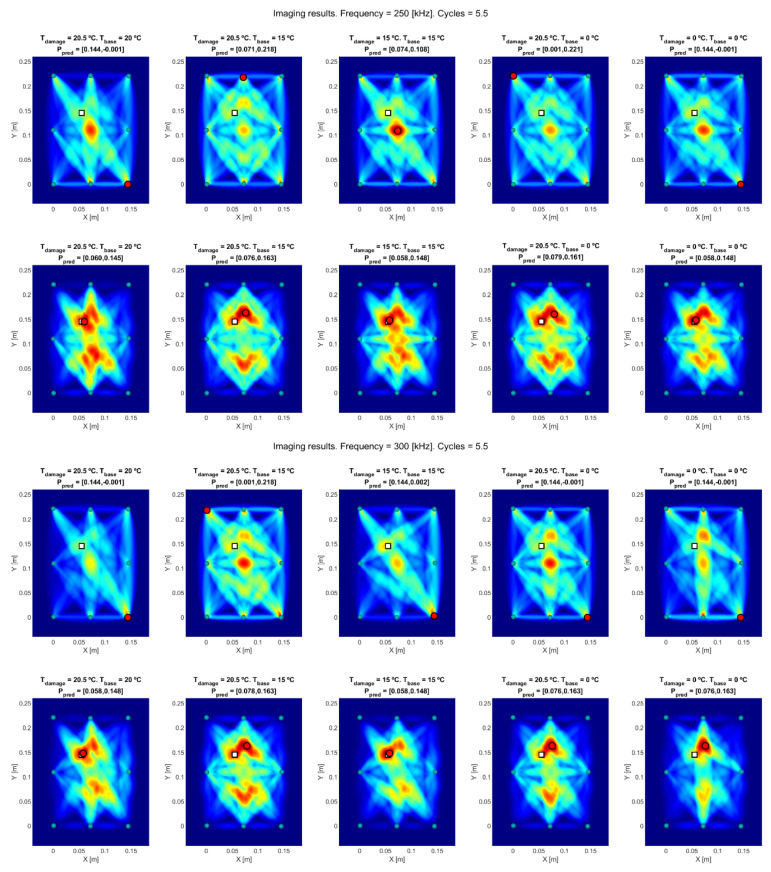

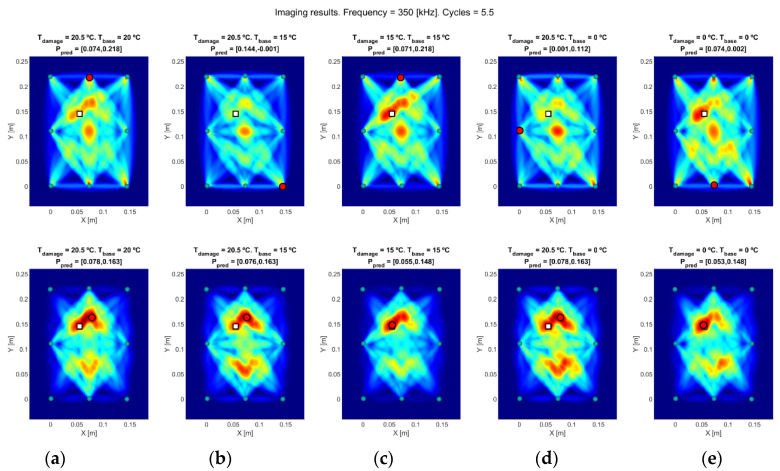
Obtained results from (**a**) real current test vs. real baseline at 20 °C, (**b**) real current test vs. real baseline at 15 °C, (**c**) modified test to 15 °C vs. real baseline at 15 °C, (**d**) real current test vs. real baseline at 0 °C, (**e**) modified test to 0 °C vs. real baseline at 0 °C. Figures at top are calculated using standard RAPID, and figures at bottom are calculated using RAPID-G. Real damage location is on white square and predicted damage location is on red circle.

The regression curves calculated previously were used to compensate the current damaged signal at $T_{c}$ = 20.5 °C, in order to compare it with the available baselines at the selected temperatures $T_{i}$ = 0 and 15 °C. In addition, and due to the availability of a very close temperature test at 20 °C, it was also used in the comparison to check the method out. Figure 16 depicts the obtained results, for three different frequencies (250, 300 and 350 kHz).

Two different testing methods (RAPID and RAPID-G) are depicted in Figure 16, divided into three groups of different excitation frequencies. The images at top show the results obtained using standard RAPID. The deviation from real damage is caused by intersected-paths masking, which provides a wrong location of the damage, either in a transducer position or the center of the distribution (where the number of intersected paths is maximum). The images at the bottom of each test’s group, obtained using RAPID-G, provide a better accuracy in the detection, since this algorithm reduces the influence of the intersection points between paths.

## 5. Discussion

This work deals with the reconstruction of Lamb wave signals, recorded at a temperature at which there is no baseline available, using a temperature at which there is pristine data available, and its effectiveness on applying it to damage detection and location using an imaging algorithm. The effectiveness of the method to obtain reconstructed signals is evaluated as the correlation coefficient is higher when the difference between temperatures is lower (Table 3), but its value decreases if the difference is high.

The analyzed method allows to use the baseline-damaged comparison, using the current temperature as input for the method, and compensating the acquired signals through the regression polynomials previously calculated. The previous results show that the temperature-dependent parameters (amplitude and phase) using Hilbert transform and least-squares minimizing process are correctly extracted by the algorithm, and the obtained polynomial curves from nonlinear regression follow the evolution of the parameters with high accuracy. The signal reconstruction process shows high increment in the correlation coefficient for every signal, particularly when the current temperature is near the reference baseline signal. Moreover, the shape of the reconstructed signals fits very well with the reference signals, especially in the first arrival wave packet (S0 mode), where the parameters were obtained from.

Regarding the imaging process, standard RAPID algorithm provided inaccurate results due to the overlapping of direct trajectories between paths, causing a high value in a location that does not match with the real position of the damage. To avoid this effect, RAPID-G was applied, providing more accurate results, as summarized in Table 4.

The previous results in Table 4 show that the calculated distance from the real damage to the calculated location is small (less than 3 cm in every case), and the improvement using RAPID-G for all cases is evident. In addition, results from Figure 16 show that the images obtained after signal reconstruction (columns (c) and (e), $T_{0}$ = 15 °C and $T_{0}$ = 0 °C, respectively) locate the damage with higher accuracy than before reconstruction (columns (b) and (d), respectively).

Summarizing, the combination of this methodology for temperature effects on Lamb waves-based SHM with RAPID-G imaging process, presents an effective approach to mitigate the drawbacks caused by EOC in a real monitoring application.

Finally, the ultimate goal of this data-driven approach is to be applied as a deep learning solution to automatically correct the undesirable effects of EOC to real applications of SHM, using Neural Networks (NN). The baseline dataset would be used to train the network, while the damaged time-domain signal at $T_{c}$ would be used as input for the NN (as well as the current temperature). The output of the NN would be the reconstructed signal to use over the optimum baseline at $T_{0}$ for damage detection, finally introduced to the imaging algorithm to locate the damage. This methodology, coupled with adaptive training of the data, would be a promising solution for deep learning SHM.

## Figures and Tables

**Table 1:** Specimen’s thermoset matrix material general characteristics.

Matrix polymer	Carbon Fiber Material	Number of Layers	Ply/Total Thickness (mm)	Stacking Sequence
M21/194/34%/T800S Unidirectional Prepreg (Hexcel)	Toray T800S	12	0.184/2.208	[+45/−45/0_2_/90/0]_S_

**Table 2:** Excitation signal characteristics.

	Amplitude (V)	Frequency (kHz)	Number of Cycles
Initial	12	250	3.5
Final		350	5.5
Step	-	50	2.0

**Table 3:** Evolution of the correlation coefficient before and after compensation.

*T*_reference_ (°C)	*T*_current_ (°C)	Path	Initial Correlation	Final Correlation	Correlation Increment
0	25	4–8	0.288	0.841	0.553
		3–5	0.004	0.725	0.721
	−10	4–8	0.862	0.967	0.105
		3–5	0.769	0.966	0.197
	35	4–8	0.093	0.737	0.644
		3–5	−0.119	0.744	0.863

**Table 4:** Distances (in centimeter) from the obtained results using RAPID algorithm (previous) and RAPID-G algorithm (final).

Frequency (kHz)	*T*_reference_ (°C)	Previous Distance to Damage (cm)	Final Distance to Damage (cm)
250	20	17.10	0.50
	15	4.16	0.42
	0	17.10	0.42
300	20	17.10	0.42
	15	16.84	0.42
	0	17.10	2.77
350	20	7.54	2.92
	15	7.47	0.30
	0	14.43	0.36

## Supplementary Materials

The dataset used in this study is available online at http://ieee-dataport.org/2543.

## References

[1] Schmerr L.W. (2016). Fundamentals of Ultrasonic Nondestructive Evaluation.

[2] Drinkwater B.W., Wilcox P.D. (2006). Ultrasonic arrays for non-destructive evaluation: A review. NDT E Int..

[3] Xiao W., Howden S., Yu L. Composite bond quality nondestructive evaluation with noncontact Lamb wave system. Proceedings of the Nondestructive Characterization and Monitoring of Advanced Materials, Aerospace, Civil Infrastructure, and Transportation IX.

[4] Farrar C.R., Worden K. (2007). An introduction to structural health monitoring. Philos. Trans. R. Soc. A Math. Phys. Eng. Sci..

[5] Balageas D., Fritzen C.P., Güemes A. (2010). Structural Health Monitoring.

[6] Cantero-Chinchilla S., Beck J.L., Chiachío M., Chiachío J., Chronopoulos D., Jones A. (2020). Optimal sensor and actuator placement for structural health monitoring via an efficient convex cost-benefit optimization. Mech. Syst. Signal Process..

[7] Wang Y., Qiu L., Luo Y., Ding R., Jiang F. (2020). A piezoelectric sensor network with shared signal transmission wires for structural health monitoring of aircraft smart skin. Mech. Syst. Signal Process..

[8] Moreu F., Li X., Li S., Zhang D. (2018). Technical specifications of structural health monitoring for highway bridges: New Chinese structural health monitoring code. Front. Built Environ..

[9] Li H.N., Ren L., Jia Z.G., Yi T.H., Li D.S. (2016). State-of-the-art in structural health monitoring of large and complex civil infrastructures. J. Civ. Struct. Health Monit..

[10] Randall R.B. (2004). State of the art in monitoring rotating machinery-part 1. Sound Vib..

[11] Randall R.B. (2004). State of the art in monitoring rotating machinery-part 2. Sound Vib..

[12] Herrmann S., Wellnitz J., Jahn S., Leonhardt S. (2014). Structural health monitoring for carbon fiber resin composite car body structures. Sustainable Automotive Technologies 2013.

[13] Barke D., Chiu W.K. (2005). Structural health monitoring in the railway industry: A review. Struct. Health Monit..

[14] Qing X., Li W., Wang Y., Sun H. (2019). Piezoelectric transducer-based structural health monitoring for aircraft applications. Sensors.

[15] Güemes A., Fernandez-Lopez A., Pozo A.R., Sierra-Pérez J. (2020). Structural Health Monitoring for Advanced Composite Structures: A Review. J. Compos. Sci..

[16] AERO—Boeing 787 from the Ground Up. https://www.boeing.com/commercial/aeromagazine/articles/qtr_4_06/.

[17] Pora J. Composite materials in the airbus A380-from history to future. Proceedings of the 13th International Conference on Composite Materials (ICCM-13).

[18] Martins T., Infante V., Sousa L., Fonseca A., Antunes P.J., Moura A.M., Serrano B. (2020). Numerical and experimental study of aircraft structural health. Int. J. Fatigue.

[19] Steinweg D., Hornung M. Integrated Aircraft Risk Analysis Framework for Health Monitoring Systems–A Case Study for Structural Health Monitoring. Proceedings of the AIAA Scitech 2020 Forum.

[20] Farrar C.R., Worden K. (2012). Structural Health Monitoring: A Machine Learning Perspective.

[21] Anton S.R., Park G., Farrar C.R., Inman D.J. On piezoelectric Lamb wave-based structural health monitoring using instantaneous baseline measurements. Proceedings of the Health Monitoring of Structural and Biological Systems 2007.

[22] Lanza di Scalea F., Salamone S. (2008). Temperature effects in ultrasonic Lamb wave structural health monitoring systems. J. Acoust. Soc. Am..

[23] Morteza T., Jan H., Steven D., Koen V. (2017). Visualization of Delaminations in Composite Structures Using a Baseline-Free, Sparse Array Imaging Technique Based on Nonlinear Lamb Wave Propagation. Acta Acust. United Acust..

[24] Fierro G.P.M., Meo M. A nonlinear ultrasonic hybrid modulation subtraction method for structural health monitoring using sparse arrays. Proceedings of the Nondestructive Characterization and Monitoring of Advanced Materials, Aerospace, Civil Infrastructure, and Transportation IX.

[25] Scalerandi M. (2016). Power laws and elastic nonlinearity in materials with complex microstructure. Phys. Lett. A.

[26] Fendzi C., Rebillat M., Mechbal N., Guskov M., Coffignal G. (2016). A data-driven temperature compensation approach for Structural Health Monitoring using Lamb waves. Struct. Health Monit..

[27] Sun H., Yi J., Xu Y., Wang Y., Qing X. (2019). Identification and Compensation Technique of Non-Uniform Temperature Field for Lamb Wave-and Multiple Sensors-Based Damage Detection. Sensors.

[28] Zhao X., Gao H., Zhang G., Ayhan B., Yan F., Kwan C., Rose J.L. (2007). Active health monitoring of an aircraft wing with embedded piezoelectric sensor/actuator network: I. Defect detection, localization and growth monitoring. Smart Mater. Struct..

[29] Azuara G., Barrera E., Ruiz M., Bekas D. (2019). Damage Detection and Characterization in Composites Using a Geometric Modification of the RAPID Algorithm. IEEE Sens. J..

[30] PiCeramic Piezoelectric Actuators, “PI_CAT128E_R3_Piezoelectric_Actuators”. https://static.piceramic.com/fileadmin/user_upload/physik_instrumente/files/CAT/PI_CAT128E_R3_Piezoelectric_Actuators.pdf?_ga=2.137927666.39786750.1550763551-149680812.1550763551.

[31] Foote P. (2013). New Guidelines for Implementation of Structural Health Monitoring in Aerospace Applications. SAE Int. J. Aerosp..

[32] Xu B., Yu L., Giurgiutiu V. Advanced methods for time-of-flight estimation with application to Lamb wave structural health monitoring. Proceedings of the 7th International Workshop on Structural Health Monitoring.

[33] Santoni G.B., Yu L., Xu B., Giurgiutiu V. (2007). Lamb wave-mode tuning of piezoelectric wafer active sensors for structural health monitoring. J. Vib. Acoust..

[34] Yuan F.G. (2016). Structural Health Monitoring (SHM) in Aerospace Structures.

[35] Fitzgibbon A., Pilu M., Fisher R.B. (1999). Direct least square fitting of ellipses. IEEE Trans. Pattern Anal. Mach. Intell..

